# A Rare Case of Hemoglobin E/Beta-Thalassemia and Systemic Lupus Erythematosus

**DOI:** 10.7759/cureus.10332

**Published:** 2020-09-09

**Authors:** Ibrahim Khamees, Ibrahim Mohammad Obeidat, Waail Rozi, Mohamed A Yassin

**Affiliations:** 1 Internal Medicine, Hamad Medical Corporation, Doha, QAT; 2 Hematology and Oncology, Hamad General Hospital, Doha, QAT

**Keywords:** systemic lupus erythematosus, hemolytic anemia, hemoglobin e/beta-thalassemia

## Abstract

Systemic lupus erythematosus (SLE) is a systemic autoimmune disease, with multisystemic involvement. Hemoglobin E/beta-thalassemia (HbE/beta-thalassemia) is the genotype responsible for approximately one-half of all severe beta-thalassemia worldwide. When beta-thalassemia and SLE coexist, SLE seems to have a more severe course. Here we report a 32-year-old female who presented with dizziness and fatigue was found to have severe hemolytic anemia with thrombocytopenia. Upon further evaluation, she was diagnosed with HbE/beta-thalassemia and SLE, which is a very rare association. In SLE patients, anemia usually results from the disease itself, but it is important to think of other coexisting conditions like thalassemia.

## Introduction

Systemic lupus erythematosus (SLE) is a multisystem chronic inflammatory disease of autoimmune etiology, with the hematologic system being affected in most cases. SLE can cause anemia in multiple ways, one of them is hemolytic anemia. It can also cause thrombocytopenia especially if associated with antiphospholipid antibody syndrome involving autoantibodies against platelets, glycoprotein IIb/IIIa or thrombopoietin receptor [1]. With that being mentioned, some patients with SLE can have another condition that can cause anemia as well. It was found that the prevalence of beta-thalassemia in patients with SLE is lower than that in general population, but if coexistence happens, SLE seems to have a more severe course [2].

Hemoglobinopathies are considered the commonest recessive monogenic disorders in the world, and they constitute a major burden on the financial and health care systems globally. Beta-thalassemia presents with either decreased (β+) or absence (β o) of the beta-globin chains in the HbA protein, which leads to buildup of extra unbound alpha-globin chains in the erythroid precursors in the bone marrow and in the red blood cells (RBCs), resulting in ineffective erythropoiesis and hemolysis. Around 1.5% of the world population are heterozygotes (carriers) of the beta-thalassemias; there is a high incidence in the Middle East, the Indian subcontinent, Southeast Asia, and Melanesia to the Pacific Islands [3].

Chronic blood transfusion (CBT) is essential in the management of individuals with beta-thalassemia major. However, it has major complications including iron overload that necessitates regular monitoring and treatment by long-term iron chelation therapy to protect the patients from endocrinopathies and cardiomyopathies, which can be fatal [4-7]. Skeletal changes may result from the expansion of the bone marrow, and development of masses from extramedullary hematopoiesis. In spite of that, CBT causes iron overload that requires monitoring and management through long-term iron chelation therapy [8].

## Case presentation

A 32-year-old female patient presented with a history of dizziness and fatigue increasing in the last few weeks. She came to the country around seven months back and mentioned having multiple blood transfusions in her home country for anemia. On examination, the patient had severe pallor, jaundice and hepatosplenomegaly.

Initial laboratory investigations are given in Table 1

**Table 1:** Initial laboratory investigations

Investigation	Result	Normal range
White blood cell count	4.5 x 10^3^/μL	4-10 x 10^3^/μL
Red blood cell count	1.7 x 10^6^/μL	3.8-4.8 x 10^6^/μL
Hemoglobin	3.1 gm/dL	12-15 gm/dL
Hematocrit	11.4%	36%-46%
Mean corpuscular volume	65.9 fL	83-101 fL
Platelet count	72 x 10^3^/μL	50-400 x 10^3^/μL
Reticulocyte %	4.2%	0.5%-2.5%
Reticulocyte index	0.46%	>2.5%
Lactate dehydrogenase	933 U/L	135-214 U/L
Haptoglobin	>10 mg/dL	30-200 mg/dL
Total bilirubin	57 μmol/L	0-21 μmol/L
Direct bilirubin	12 μmol/L	0-5 μmol/L
Ferritin	2,519 μg/L	12-160 μg/L
Iron	43 μmol/L	6-35 μmol/L
Fe saturation	90%	15%-45%
Direct antiglobulin test	+2 for immunoglobulin G	
Elution test	Negative	

**Figure 1:**
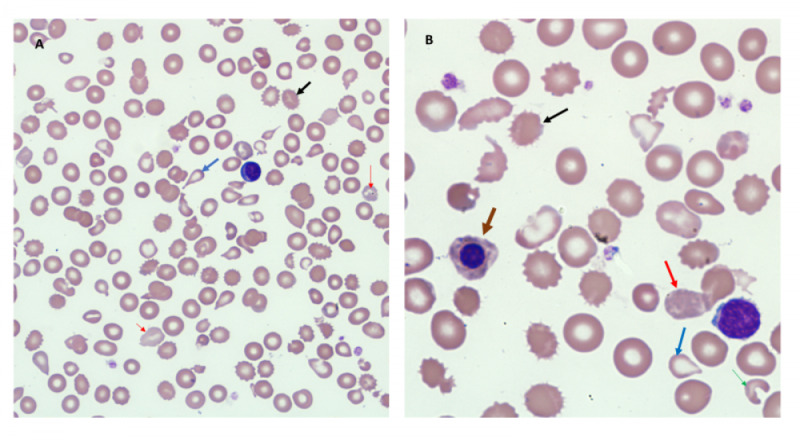
Peripheral blood smear Image A is low power view. Image B is high power view. Both images show hypochromic microcytic anemia with scattered polychromatic cells (red arrow), Nucleated red blood cells (brown arrow) and significant anisopoikilocytosis including many scattered tear drops (blue arrow), ovalocytes, schistocytes (green arrow), burr cells (black arrow) and some microspherocytes.

Peripheral smear (Figure 1) showed moderate hypochromic microcytic anemia with scattered polychromatic cells, many nucleated RBCs and significant anisopoikilocytosis, including many scattered tear drops, ovalocytes, schistocytes, irregularly contracted cells and some microspherocytes.

Kidney function, international normalized ratio and fibrinogen were all normal. Ultrasound of the abdomen was done, which was positive for hepatosplenomegaly. The patient was resuscitated with packed RBC transfusion and admitted. With the history of multiple blood transfusions in the past, the iron overload (probably due to multiple blood transfusions) and the low mean corpuscular volume (MCV), hemoglobin electrophoresis was sent and it showed the findings in Table 2, based on that the patient was diagnosed with HbE/beta-thalassemia.

**Table 2:** Hemoglobin electrophoresis

Hemoglobin type	Percentage
Hb A	00
Hb A2	2.0
Hb F	25
Hb S	00
Hb E	73

Autoimmune workup revealed positive antinuclear antibody (ANA), anti-double-stranded DNA (anti-ds-DNA) and low complement protein 3 (C3), so the patient was diagnosed with SLE and started on hydroxychloroquine. The patient was hospitalized for five days, during which Hb picked up to 9.9 gm/dL and platelets count decreased to 38 x 10^3^/μL, and was discharged with a plan to follow complete blood cell (CBC) in the clinic after two weeks. However, upon repeating CBC after two weeks, the patient had anemia with Hb of 6.9 mg/dL, white blood cell (WBC) of 3.7 x 10^3^/μL, absolute neutrophilic count of 2.2 x 10^3^/μL and platelet count of 12 x 10^3^/μL, so she was admitted again. She complained of easy fatigability and exertional shortness of breath, but no apparent bleeding from any site. On examination, she had pallor, hepatosplenomegaly but no skin rash.

Her lab investigations that time also revealed hemolytic anemia with low reticulocyte index 0.55 and severe thrombocytopenia 12 x 10^3^/μL, increased (anti-ds-DNA) titer, low C3 and normal kidney function test. She was managed as a case of SLE flare causing thrombocytopenia and anemia complicated by thalassemia, for which she was started on oral prednisolone 1 mg/kg which was changed to intravenous methylprednisolone 10 mg/kg, along with platelets, fresh-frozen plasma and packed RBC transfusions. She was also started on deferasirox (Jadenu) 720 mg daily due to iron overload. In the next few days, the platelet count improved to 47 x 10^3^/μL and her Hb stabilized around 9 mg/dL, so she was discharged on oral prednisolone with a follow-up after one week to follow CBC. In the follow-up, her platelet count was 119 x 10^3^/μL and her Hb was 9.3 mg/dL, so prednisolone was tapered with close follow-up in rheumatology and hematology clinics.

## Discussion

We described a patient who has HbE/beta-thalassemia double heterozygosity, admitted with severe anemia and thrombocytopenia, found to have SLE. The association between beta-thalassemia in general and SLE is rare, and it is rarer in HbE/beta-thalassemia based on the reported case all over the world. However, near the beta-globin locus at 11p15.5, there are specific immunity genes, which match with the autoimmunity susceptibility [9].

Hematologic abnormalities are common in SLE, and all three blood cell lines can be affected. Anemia of chronic disease is the most common type of anemia in SLE patients, autoimmune hemolytic anemia is relatively rare and severe thrombocytopenia is rare as well [10].

In general, HbE/beta-thalassemia is a thalassemia syndrome of intermediate severity with a diverse clinical spectrum. Compared to a normal individual, HbA is replaced by HbE and HbF in patients with HbE/beta-thalassemia. HbE constitutes between 30% and 70% of the hemoglobin with the rest HbF. In some cases of HbE thalassemia as HbE beta+ thalassemia, different mutations lead to variable amounts of HbA, which causes variable levels of disease severity [11].

In one study that included 177 patients with SLE, there were 17 patients with beta-thalassemia. This study concluded that the prevalence of beta-thalassemia in patients with SLE seems to be lower than that in the general population. However, when the two conditions coexist, SLE seems to have a more severe course. The incidence of complications was more severe in SLE with beta-thalassemia. However, the reason behind that was not clear. It could be related to low complement 3 and complement 4, and/or due to presence of anti-SSA autoantibodies (anti-Sjögren's syndrome-related antigen A autoantibodies, also called anti-Ro antibodies). Also, higher occurrence of atherosclerotic events and loss of the ability to bind immune complexes have been described in beta-thalassemia which could be related to a more severe disease [2].

In one literature review, the authors highlighted the diagnostic difficulty of SLE in patients with other type of hemolytic anemia which is sickle cell disease (SCD). They attributed that to the similar manifestations of both diseases, including anemia and skeletal pain. They concluded that certain features might alert the physicians to the possibility of both diseases coexistence like joint pain resistant to usual analgesic measures, presence of proteinuria or presence of leukopenia and thrombocytopenia in SCD patients [12].

Many cases reported association of beta-thalassemia trait and SLE, but upon reviewing the literature we found only one case about HbE/beta-thalassemia and SLE disease [13]. In our case, we faced more severe presentation in the hematological system. Meanwhile for the patient who was diagnosed previously with thalassemia and developed worsening in her hematological cell lines, it is worthy to investigate for possible other coexisting causes. Peripheral smear finding and autoimmune antibody can give us clue about the underlying cause and the need for further testing.

## Conclusions

The association between beta-thalassemia in general and SLE is rare, and it is rarer in HbE/beta-thalassemia and SLE based on the reported case all over the world. More severe symptoms can present in concomitant disease. This warrants early suspicion and diagnosis of other diseases that might worsen the primary disease.
